# Quality and value of intensive care discharge summaries for general practitioners

**DOI:** 10.1186/cc11127

**Published:** 2012-03-20

**Authors:** F Daruwalla, FJ Lamb, CA Mearns

**Affiliations:** 1 Surrey and Sussex Healthcare NHS Trust, Redhill, UK

## Introduction

Good communication between healthcare professionals is required to provide continuity of care for patients being discharged from the ICU [1]. It is our unit's practice to send a copy of a patient's computerized ICU discharge summary to both the hospital team with ongoing responsibility and to their general practitioner (GP). The aim of this study was to establish and compare the quality and value of the summaries as judged by ICU doctors and GPs.

## Methods

Discharge summaries for patients admitted in July 2011 were obtained from the ICU WardWatcher^® ^database. These were scored independently by two ICU consultants and a trainee doctor using a predefined rating scale. The GPs were sent postal questionnaires regarding their perceptions of the quality and value of the summaries. A comparison was made between the ratings made by the ICU team and the responses to the GP questionnaires.

## Results

Sixty patients were admitted during the study period. All 60 summaries were independently rated by three ICU doctors and good inter-rater reliability was demonstrated (Cronbach's α = 0.89). There was a strong correlation between the ratings given by the ICU consultants and the trainee doctor (Spearman's = 0.91). Twenty-eight per cent achieved an acceptable score of 6 out of 10 or greater (median score 5, interquartile range 3 to 6). Fifty-four postal questionnaires were sent to GPs and 36 were returned (response rate 67%). Seventy-six per cent achieved an acceptable score of 16 out of 25 or greater (median score 18, interquartile range 16 to 25). Sixty-nine per cent of GPs found the discharge summary helpful and 86% wanted to be sent this type of summary in future. Correlation between the ICU team rating and the GP score for the summaries was weakly positive (Spearman's = 0.15).

## Conclusion

Although only 28% of discharge summaries achieved an acceptable or higher rating from the ICU team, GPs valued the majority of discharge summaries issued by our ICU. Further research is needed to explain the difference between ICU doctors' perception of discharge summary quality and the value provided by them to GPs.
